# A Portable Styptic

**Published:** 1862-10

**Authors:** 


A Portable Styptic.—For the preparation of a convenient styptic, it is recommended by the *Monitaur des Scienees Médicales* to soak amadon or German tinder in a solution of perchloride of iron of a density of about 1·250. It should then be dried in the sun, and rubbed between the hands to restore its suppleness and porosity. Small pieces applied to leech bites soon stop their bleeding. They may be held in their places by strips of plaster.

